# Optimization of LDO-Pectin Synthesis Conditions for the Removal of Metals from Wastewater: A Comparison of Response Surface Methods and Taguchi Approaches

**DOI:** 10.3390/polym15183778

**Published:** 2023-09-15

**Authors:** Ava Mohrazi, Reza Ghasemi-Fasaei, Amin Mojiri, Sedigheh Safarzadeh Shirazi

**Affiliations:** 1 Department of Soil Science, School of Agriculture, Shiraz University, Shiraz 71348-14336, Iran; 2 Envirowise Research Associate, Christchurch 8053, New Zealand

**Keywords:** RSM, modified LDO, heavy metal, pectin

## Abstract

With the continuous growth of industrialization, the presence of heavy metals (HMs) in the environment has become a critical issue, necessitating cost-effective and efficient techniques for their removal. The present study aimed to determine the optimal preparation conditions for synthesizing pectin (PC) as a polymer sorbent, combined with Magnesium (Mg) Aluminum (Al) layered double oxides (LDOs), using a fast and facile co-precipitation method. Both the response surface method (RSM) and the Taguchi method were employed to optimize the influence of key independent variables, including the molar ratio of cations Mg:Al, the ratio of pectin to LDO, and the temperature for removing multiple elements from wastewater. The results indicated that RSM is more accurate and examines more interactions, while Taguchi reduces the number of tests and is more economical than RSM. However, both statistical methods showed good potential for predicting the adsorption capacity (Qe) of HMs. The optimal preparation conditions were identified as a molar ratio of 3:1, a ratio of pectin to LDO of 7% *w*/*w*, and a temperature of approximately 600 °C. In conclusion, the application of RSM and Taguchi approaches was found to be feasible and effective in optimizing the preparation conditions of modified LDO, which can be utilized as a potential adsorbent for removing multiple elements from wastewater.

## 1. Introduction

Industrial wastewater containing toxic heavy metals (HMs) has led to severe environmental issues in recent years due to rapid industrialization [1]. HMs contaminate water, infiltrating the food chain and posing risks to organisms. Consequently, it is imperative to develop an effective and straightforward method for eliminating HMs from aqueous solutions [2]. Various techniques like biodegradation [3], photo-degradation [4], and chemical oxidation [5] have been employed for HMs removal from wastewater. However, these methods come with limitations, such as high energy consumption [6]. An expeditious, cost-effective, and efficient approach for HM removal is through adsorption. Utilizing adsorbents that are both economical and environmentally friendly can significantly enhance the efficiency of the adsorption process [7].

Layered double hydroxides (LDH), anionic clay adsorbents with layered structures, represent an excellent choice for eliminating pollutants from aqueous solutions due to their favorable interlayer anion exchangeability, non-toxic properties, exceptional surface area, and ease of preparation [8,9]. Some researchers propose that layered double oxide (LDO), the calcined byproduct of LDH, possesses a larger surface area and greater adsorption capacity compared to LDH [1]. The adsorption capacity (Qe) values for Magnesium (Mg) Manganese (Mn)-LDH and MgMn-LDO are 5.635 and 8.234 (mmol g^−1^), respectively, suggesting that the active sites on MgMn-LDO are more abundant than those on MgMn-LDH [10]. As a result, LDO could serve as a valuable sorbent for remediating both organic and inorganic pollutants.

The limitation of relying solely on LDHs is their lack of stability over extended periods, rendering them non-regenerable. To enhance stability, larger recalcitrant material particles can support LDH, including materials derived from agricultural waste like biochar and activated carbon [9]. Encouraging results have been demonstrated by LDH-biochar composites in HM removal [11]. The utilization of zinc (Zn) and Aluminum (Al)-LDH/biochar led to a 44% increase in pollutant removal compared to the use of ZnAl-LDH alone [12,13], successfully introducing an innovative adsorbent, LDH-biochar, to bolster the adsorption capacity and prevent LDH disintegration.

Green adsorbents derived from agricultural waste find application as economical and eco-friendly sorbents for removing HMs from aqueous solutions [14]. Pectin (PC), a polymer obtainable from most plants, is a green adsorbent utilized for wastewater cleanup. Its composite with other adsorbents, such as chitosan, holds significant potential to enhance adsorption capacity and stability [15]. The licorice plant (*Glycyrrhiza glabra* L.), a perennial herb with valuable components, has garnered attention in the food and pharmaceutical industries in Iran and other nations [16]. Due to its medicinal and commercial value, illicit cultivation of this plant is rising, leading to the generation of waste materials. However, no study has explored the utilization of licorice waste as a foundational material for adsorbents. Thus, biomass wastes like licorice pomace present a viable option for synthesizing PC to address cost and environmental concerns.

The capacity of commonly used LDO and modified LDO in adsorption methods are influenced by various factors, including calcination temperature, molar ratio (M^+2^/M^+3^), weight, and the source of the bioabsorbent [17]. Comparing the effects of these factors necessitates a broad range of experiments, which can be time-consuming, expensive, and challenging. Taguchi and Response Surface Methodology (RSM) are potent statistical-mathematical techniques for modeling, optimizing, designing experiments, and analyzing the impact of independent operational factors and their interactions. These approaches can help reduce the number of experiment runs and associated costs [18,19]. However, as far as the authors are aware, there is limited research comparing these two statistical methods for optimizing LDO-pectin synthesis conditions in metal removal from wastewater. Achieving the highest removal efficiency for each HM demands specific LDO with well-defined preparation conditions. Yet, no study has focused on determining the optimal and economic conditions for LDO synthesis and the interaction effects of the three most crucial factors on its sorption capacity.

This study encompassed the characterization of modified adsorbents, assessment of the removal efficiency of HMs from aqueous solutions, determination of adsorption capacity, and exploration of adsorption mechanisms. The results of this study hold potential for the development of efficient and environmentally friendly adsorption materials designed to eliminate HMs from aqueous solutions.

The aim of this study is to achieve the optimal conditions for synthesizing modified LDO (LDO-pectin/LDO-PC) adsorbents using RSM and Taguchi optimization methods. Initially, the experiment was conducted following the arrangement suggested by RSM, and subsequently, the same arrangement was used to design the Taguchi test. Initially, the removal efficiency (RE) (%) of nickel (Ni), zinc (Zn), cadmium (Cd), and lead (Pb) by all prepared sorbents was determined. Then, the optimal and less effective adsorbents (undesirable) were selected, and their characteristics were measured. Finally, kinetic and isotherm tests were conducted using the optimal sorbent. This study also represents the first investigation into modified LDO (LDO-PC) synthesis and its optimal conditions.

## 2. Materials and Methods

### 2.1. Materials

Licorice pomace was collected from Fars Osareh Iranian Industries Factory located in Fars province. All chemical reagents (AlCl_3_, MgCl_2_, Zn(NO_3_)_2_·6H_2_O, Ni(NO_3_)_2_·6 H₂O, Cd(NO_3_)_2_·4H_2_O, Pb(NO_3_)_2_, HCl, NaOH, and NaCl) in the present research were analytically pure 98% and purchased from Merck company.

### 2.2. Preparation of Different LDH, LDO, and LDO/LDH Supported by Pectin

The production of various adsorbents involved four stages. In the first stage, pectin was synthesized from Licorice pomace through a microwave-assisted method [20]. The licorice pomace was washed, air-dried, and then sieved. Then, 10 g Licorice pomace powder was added to 250 mL distilled water, and the pH was reached to 1.5 with HCl (2N). Then, the suspension was irradiated with a microwave for 3 min (180 s), filtered, and centrifuged. Then, the supernatant was mixed, washed with 95% ethanol, and dried to obtain pectin. In the second stage, Mg–Al LDH was prepared using a facile, co-precipitation, and simple method. Mg and Al as nitrate source salt in various ratios molar (2:1, 3:1, and 4:1 M^+2^:M^+3^) were mixed in distilled water and stirred for 30 min. Then, sodium hydroxide (2 mol L^−1^) was added dropwise until the pH reached 9.0, then kept for 24 h at 65 RT, centrifuged, filtered, and washed with distilled water to obtain LDH. In the third stage, LDH was calcined in a muffle furnace at different temperatures (0, 300, and 600 RT) for 3 h to produce LDO [10]. In the fourth stage, LDH or LDO was mixed with pectin in different weight ratios (0, 5, and 10% *w*/*w*) to synthesize LDH-PC or LDO-PC. Pectin was sonicated in distilled water for 25 min, and LDH or LDO was added to form a mixed solution. The pH was adjusted to 10–11, stirred for 24 h, and filtered to obtain LDH-PC or LDO-PC. All three materials were sieved through a 0.45 μm sieve and stored for further steps.

### 2.3. Experimental Design

#### 2.3.1. RSM

Using Box–Behnken Design (BBD), the optimal molar ratio of cation, temperature, and ratio of pectin to LDO were determined. Fifteen samples of various adsorbents were prepared based on the BBD experimental design and were subsequently used in the adsorption process with 100 mg L^−1^ solutions of four HMs as nitrate sources (Zn, Pb, Ni, and Cd). The RSM was employed, and a quadratic model, as presented in Equation (1), was developed to predict the best preparation conditions and their interactions.

(1) $$Y=b_{0}+b_{1}A+b_{2}B+b_{3}C+b_{12}AB+b_{13}AC+b_{23}BC+b_{11}A^{2}+b_{22}B^{2}+b_{33}C^{2}$$

where Y is the predicted response value; *A*, *B*, and *C* refer to the molar ratio of cation (Mg:Al), ratio of pectin to LDO, and temperature, respectively. The terms AC, BC, and AB showed the interaction of operation factors. C^2^, B^2,^ and A^2^ showed variables in square form. b_13_, b_12,_ and b_23_ were the interaction coefficients between the variables. b_1_, b_2_ and b_3_ were linear coefficients.

The total number of experiments run was calculated as follows:

N = 2B (B − 1) + D_0_ (2)

where D_0_ and B were the number of central point and operation factors, respectively. Utilizing Design-Expert (version 10.0), the experimental setup and data were examined. Selected concentrations and levels of factors have been referred to in previous research [21,22,23]. The level and range of operation factors and the design of the experiment are listed in Table 1 and Table 2.

#### 2.3.2. Taguchi Method

In the Taguchi method, just 9 runs are required to reach the optimal levels of each independent variable (Table 3), but in the traditional method, a larger number is required, which is practically time-consuming. Taguchi uses the signal-to-noise ratio (S/N) in measurable amounts of qualitative characteristics according to the aim of the tests, which is calculated by Equation (3):

(3) $$S/N=-10\log\;[\frac{1}{n}\Sigma {\left(\frac{1}{Y}\right)}^{2}]$$

where n and y are the number of experiments and responses, respectively.

Utilizing Design-Expert (version 10.0) and Minitab (version 17), the experimental setup and data were examined.

### 2.4. Adsorption Experiments

The efficiency and properties of all sorbents produced through RSM and Taguchi were evaluated in adsorption experiments. A total of 0.02 g adsorbent (1 g L^−1^) and 20 mL of four HMs (Zn, Pb, Ni, and Cd as nitrate source) solution (100 mg L^−1^ concentration of HMs) were added in Centrifuge Tubes. The solution was shaken for 180 min, then centrifuged and filtered, and the values of HMs concentrations were determined by an Atomic absorption spectrophotometer (AAS) (Shimadzu, AA 670G, and Japan).

The adsorption capacity (Q_e_) and percentage removal efficiency (RE %) of 4 studied HMs were calculated by Equations (4) and (5)

(4) $$Q_{e=}\frac{{(c}_{0-}c_{e})v}{m}$$

(5) $$\mathrm{RE}=\frac{C_{0}-C_{e}}{C_{0}}\times 100$$

where, C_0_ and C_e_ (mg L^−1^) are the initial and final concentrations of HMs, and V (L) and M (g) represent the volume of the solution and sorbent dose, respectively. In the adsorption experiment, the wavelengths were 213, 217, 228, and 232 nm for Zn, Pb, Cd, and Ni, respectively.

### 2.5. Characterization

After preparing the sorbents through RSM and Taguchi methods and evaluating their efficiency, optimal (modified LDO) and undesirable (LDH) sorbents were selected for further characterization. Scanning electron microscopy (SEM), energy-dispersive X-ray spectroscopy (EDX), Fourier transform infrared (FTIR), and pH point of zero charges (pH pzc) measurements were employed to characterize and differentiate the modified LDO and LDH sorbents.

#### 2.5.1. Morphology

Fourier transform infrared spectroscopy (FT-IR) (Tensor II, Germany) and SEM with an attached energy dispersive x-ray spectrometer (EDX) (TESCAN-Vega 3, Czech Republic) were used to characterize the functional groups, morphology, and elemental compositions of the modified LDO and LDH sorbents produced. The specific surface area of sorbents was determined by the multi-point Brunauer–Emmett–Teller (BET) technique.

#### 2.5.2. Determination of pH at the Point of Zero Charges (pH_pzc_)

A total of 0.3 g of sorbents was added to 50 mL of solution containing NaCl (0.01 M), and the initial pH (pHi) value was adjusted with either NaOH (2 M) or HCl (2 M) to reach a range of (3–11). After equilibrating for overnight, the final pH (pH_f_) was measured [24]. The pH pzc of the sorbent was obtained from the graph that ∆pH (pHf − pHi) was plotted versus pHi. The pH at which ∆pH = 0 was considered as the pHpzc.

#### 2.5.3. Reusability of the Modified LDO and LDH Sorbents

To investigate the reusability of the modified LDO and LDH sorbents, the adsorption process was repeated four times. The reusability of sorbents, after adsorption of HMs, was performed through filtration, rinsing with distilled water and ethanol several times, and drying at 105 RT. Then, the adsorption process of HMs onto the recovered sorbent was performed [25], and the RE (%) was assessed using Equation (5) to calculate the sorbent’s performance in terms of reusability.

### 2.6. Kinetic and Isotherm Models

To evaluate the adsorption mechanism of modified LDO sorbent, the isothermal adsorption and kinetics study was researched.

Four kinetic models (Equations (6)–(9)) and two isotherm models (Equations (10) and (11)) were employed to evaluate the patterns of HMs-adsorption at various contact/react times (5–180 min) and initial concentration (10–100 mg L^−1^). Also, these models were assessed using the R^2^ analysis term and standard error of the estimate (SE) (Equation (12)) in order to determine the kinetic and isotherm model’s best fit to the experimental data.

(6) $$\frac{t}{Q_{t}}=\frac{1}{k_{2}Q_{e}^{2}}+\frac{t}{Q_{e}}\;(\text{pseudo-second-order})$$

Ln (q_e_ − q_t_) = Lnq_e_ − k_1_t (pseudo-first-order) (7)

(8) $$Q_{t}=k_{3}t^{1/2}+c\;(\text{intra-particle diffusion})$$

(9) $${\;Q}_{t}=\alpha +\beta Lnt\;(\text{simple elovich models})$$

InQe = In K*_F_* + 1/n InC_e_ (Freundlich) (10)

(11) $$\frac{Q_{e}}{C_{e}}=k_{L}Q_{e}+Q_{max}k_{L}\;(\mathrm{Langmuir})$$

(12) $$\mathrm{SE}=\sqrt{\left\lfloor \frac{\sum {\left(Qm-Q*\right)}^{2}}{n-2}\right\rfloor }$$

where Q_e_ (mg g^−1^), Q_t_ (mg g^−1^), Q max (mg g^−1^), Qm, Q*, and Ce (mg L ^−1^) represent adsorption capacity at equilibrium time, time (t), maximum adsorption capacity, adsorption capacity measured, adsorption capacity calculated at time t and concentration HMs at equilibrium time, respectively. The rate constant of pseudo-first order, pseudo-second order, and intraparticle diffusion are k_1_ (min^−1^), k_2_ (g mg^−1^ min^−1^), and k_3_ ((mg g^−1^)min^0.5^). For the thickness of the intraparticle diffusion boundary layer, C (mg g^−1^) is a constant. Also, 1/n, K_L_ (L mg^−1^), and K_F_ (mg g^−1^) are the Freundlich adsorption intensity parameter, Langmuir constant, and Freundlich constant, respectively, and finally, n is the number of observations.

## 3. Results and Discussion

### 3.1. RSM and Taguchi Model Analysis

The experiments were optimized using the BBD (Table 4). Quadratic regression model equations for Qe of Zn, Cd, Ni, and Pb are given as Equations (13)–(16).

Qe _Zn_ = 99.94 + 0.61A + 3.83B + 4.05C − 0.34AB − 0.021AC − 1.97A^2^ − 2.23 B^2^ − 2.62C^2^ (13)

Qe _Cd_ = 98.69 + 2.29A + 2.66B − 4.25AB − 3.51B^2^ (14)

Qe _Pb_ = 99.59 + 0.39A + 2.79B + 2.68C − 2.52BC − 2.79A^2^ − 3.12B^2^ (15)

Qe _Ni_ = 98.20 + 1.38A + 3.08B + 1.94C − 1.62B^2^ (16)

Initially, significant parameters were retained, while non-significant parameters were eliminated. The expression above illustrates the synergistic effects of factors indicated by a positive sign and antagonistic effects indicated by a negative sign. It is observable that the molar ratio, temperature, and the ratio of pectin to LDO had a synergistic impact on adsorption capacity.

The maximum values for Qe (adsorption capacity) and RE (removal efficiency) were 99.99 mg g^−1^ and 99.99%, respectively, corresponding to run 12, which involved Zn (with a molar ratio of 3, ratio of pectin to LDO 10 *w*/*w*%, and a temperature of 600 °C). Conversely, the minimum Qe and RE were 83.30 mg g^−1^ and 83.30%, respectively, associated with run 1, which pertained to Cd (with a molar ratio of 2, ratio of pectin to LDO 0 *w*/*w*%, and a temperature of 300 °C).

Based on these results, an increase in the molar ratio led to an enhanced adsorption capacity, as elevated cationic molar ratios contributed to an increased specific surface area (SSA) [26]. Upon comparing all runs, it becomes evident that the composite of LDH or LDO with pectin exhibits a higher adsorption capacity than LDH or LDO alone. A previous study [27] reported that LDO composite with a bio adsorbent showcased heightened adsorption capacity due to the presence of more functional groups and increased SSA.

According to Table 5 and Equations (17)–(20), based on the Taguchi method, the ratio of pectin to LDO and temperature had a positive effect on Qe of Zn, which is in accordance with the RSM results.

Qe _Zn_ = 79.10 + 0.06 A + 4.74 B + 3.36 C (17)

Qe _Cd_ = 92.06 − 0.09 A + 2.33 B − 0.51 C (18)

Qe _Pb_ = 90.54 − 0.02 A + 2.81 B + 0.09 C (19)

Qe _Ni_ = 88.73 + 1.50 A + 1.46 B + 0.14 C (20)

#### 3.1.1. Analysis of Variance (ANOVA)

Based on Tables S1 and S2, the variances in the experimental results were analyzed using a quadratic regression model derived from the experiment’s data. ANOVA is employed in both the RSM and Taguchi models to evaluate the significance of differences between factors and the appropriateness of the model predictions compared to the experimental outcomes. The model is deemed suitable and valuable when its *p*-value is less than 0.05 (indicating significance) and its lack of fit is greater than 0.05 (indicating insignificance) [28]. With smaller *p*-values and higher F-values, all models demonstrated a favorable potential for predicting the Qe of HMs. The R^2^ values for the QeZn model based on RSM and Taguchi were 0.99 and 0.77, respectively. These results indicate that RSM exhibits superior predictive power and accuracy compared to the Taguchi method.

For instance, in the case of Zn, based on the *p*-values in Table S1, factors A, B, C, AB, and AC are significant (*p* < 0.05), while factor BC is non-significant (*p* > 0.05). The influence of the three factors on the Qe of LDO for the studied HMs (excluding Zn) was as follows: ratio of pectin to LDO > temperature > molar ratio. However, for Zn, the order of influence was temperature > ratio of pectin to LDO > molar ratio. Additionally, the impact of interaction factors was ranked as follows: AB > AC.

However, diverse outcomes were obtained through the implementation of the Taguchi statistical method. The ratio of pectin to LDO and temperature exhibited a positive and significant effect on the Qe of Zn, while other factors and their interactions demonstrated no significant impact. Moreover, in line with the RSM method, the Taguchi approach also identified the ratio of pectin to LDO as the most influential factor.

**Figure 1:**
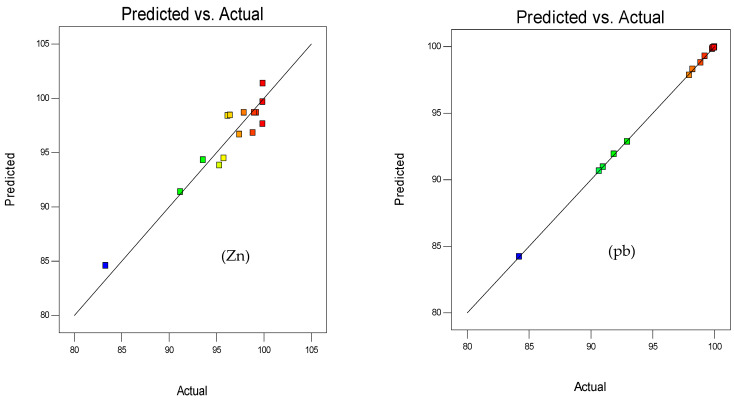

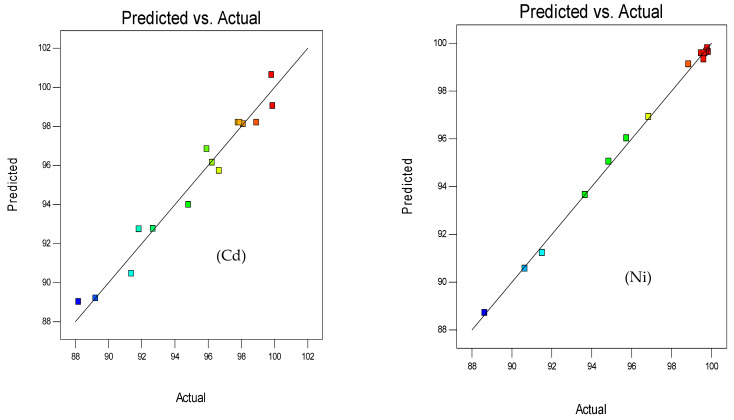
Relationship between actual and predicted data using the RSM method. Color poin by value of adsorption capacity.

**Figure 2:**
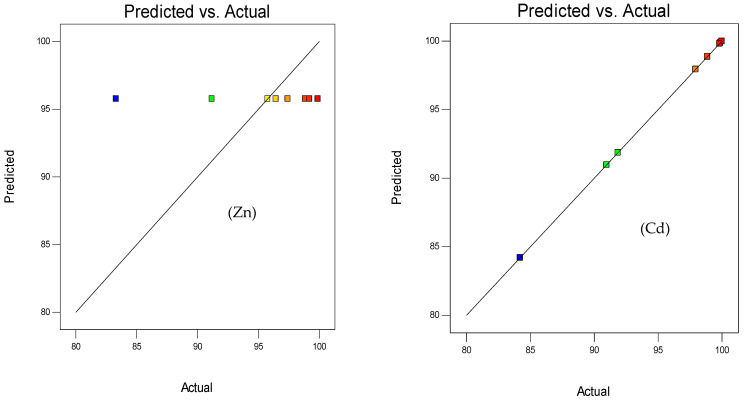

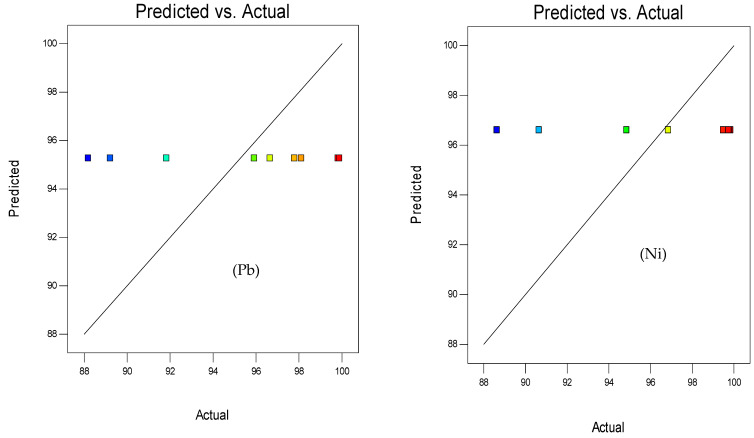
Relationship between actual and predicted data using the Taguchi method. Color poin by value of adsorption capacity.

Referring to relevant studies, when all data points lie along the line, the model is considered adequate. Figure 1 and Figure 2 indicate this adequacy by all points aligning along the diagonal between the predicted and actual values.

#### 3.1.2. Response Surface 3D Plots

**Figure 3:**
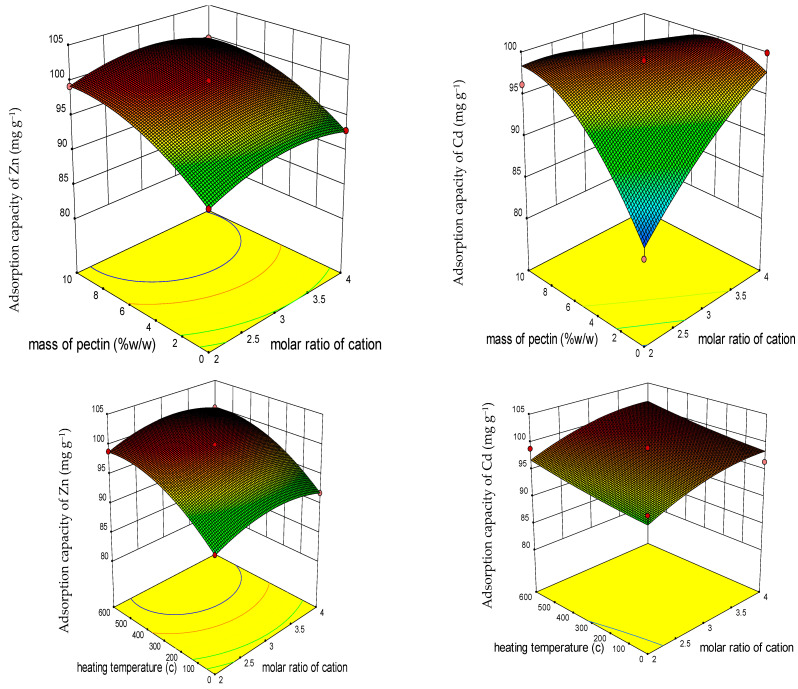

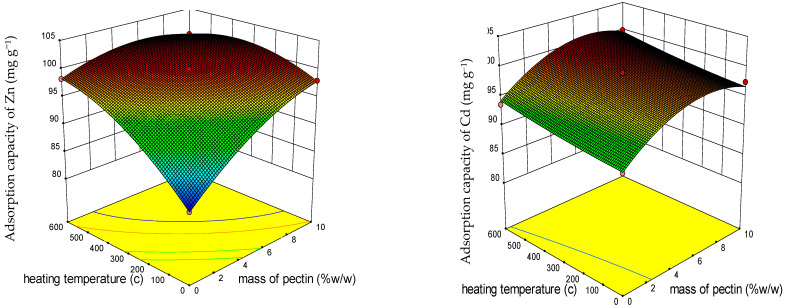
RSM three-dimensional (3D) response surface diagram of factor interactions impacting adsorption capacity (mg g^−1^) of adsorbent for Zn and Cd.

**Figure 4:**
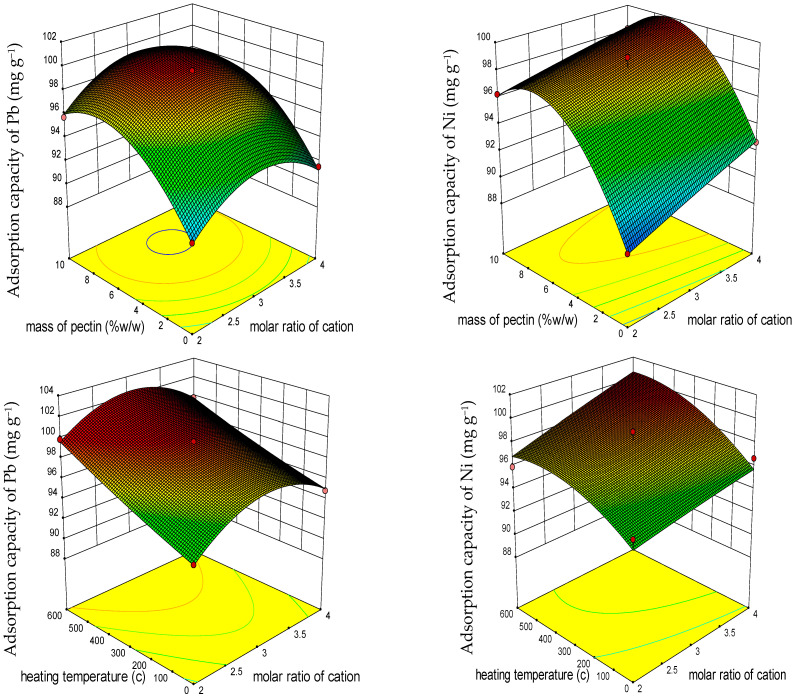

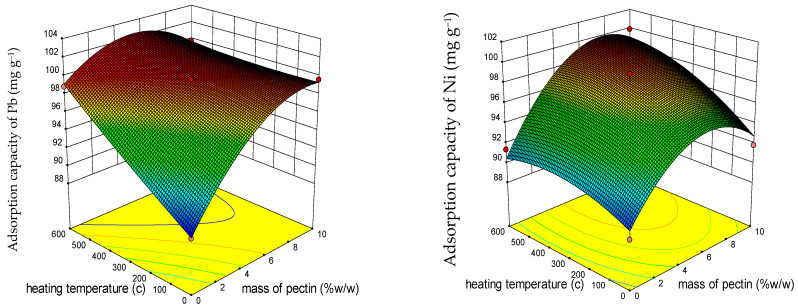
RSM three-dimensional (3D) response surface diagram of factor interactions impacting adsorption capacity (mg g^−1^) of adsorbent for Pb and Ni.

The diverse effects of each individual factor (molar ratio of cation, ratio of pectin to LDO, and temperature) and their interactions on the responses (Q_e_ of the sorbent for the studied HMs) can be depicted through the 3D response surface plots (Figure 3 and Figure 4).

Based on the results obtained from the 3D plots, the impact of both individual factors and the interactions among the three factors is significant in elucidating the Q_e_ of the sorbent in its capacity to remove HMs.

**Figure 5:**
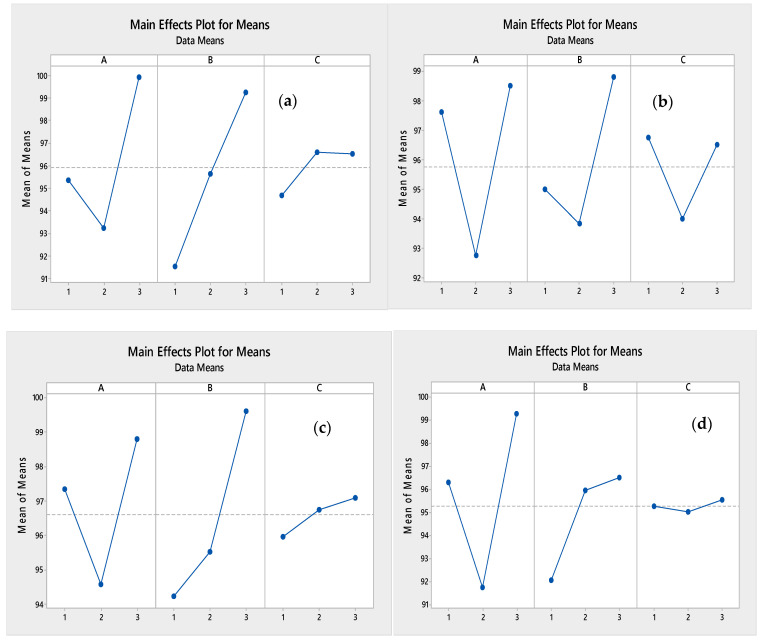
Signal-to-noise ratios for Qe of Zn (**a**), Cd (**b**), Pb (**c**), and Ni (**d**) based on the Taguchi method.

Based on the results depicted in Figure 3 and Figure 4 derived from the RSM findings, it is evident that an increase in the molar ratio of cation and the ratio of pectin to LDO led to a substantial enhancement in the Qe of the adsorbent for Zn and Ni. These outcomes were consistent with those obtained through the Taguchi method (Figure 5). The highest Q_e_ value (99.99 mg g^−1^ for Zn) was observed at a molar ratio of approximately three and a ratio of pectin to LDO of 10% *w*/*w*. Notably, for Zn, elevating the temperature had a more pronounced impact on improving the Q_e_ of the adsorbent compared to increasing the molar ratio. In contrast, for Cd and Pb, an increase in the ratio of pectin to LDO and the molar ratio initially led to a significant increase in the Q_e_ of the sorbent, which then declined. As illustrated in Figure 3, with the temperature increment, the Q_e_ of the adsorbent for Cd^+2^ remained relatively stable, signifying a resonance, as supported by the ANOVA.

In comparison to BBD–RSM, the ranking of operational parameters in terms of their contribution to response values can also be achieved using the Taguchi method. The Taguchi method also recommends its utilization for screening input variables during the initial phases of process investigation. In this current study, three factors were chosen, and it is evident that the effectiveness of each parameter is closely linked to the type of metals. For all factors, the top-ranking parameter was consistent between both methods, while the second and third rankings differed (refer to Table 6).

#### 3.1.3. Response Surface Optimization

To attain the highest Qe (mg g^−1^) using the RSM method, the optimal conditions for the molar ratio of cation, the ratio of pectin to LDO, and the temperature were determined as 3.012, 577 RT, and 6.98 (*w*/*w*%), respectively. Remarkably, these results closely mirror the optimal levels suggested by the Taguchi method. These optimal synthesis conditions for the sorbent differ from the preparation conditions used in prior research for other adsorbents. This finding underscores the necessity of distinct synthesis conditions for sorbents when addressing the remediation of various types of HMs.

Verification tests were conducted under optimal conditions to validate the accuracy of the predicted values (see Table 7). According to the data in Table 6, the actual Qe values for Zn, Cd, Pb, and Ni were 99.43, 99.98, 99.42, and 99.40 mg g^−1^, respectively. In comparison, the predicted Qe values for Zn, Cd, Pb, and Ni were 101.51, 100.86, 101.96, and 99.91 mg g^−1^, resulting in percentage errors of 2.41%, 1.30%, 2.73%, and 1.4%, respectively. However, using the Taguchi method, the percentage error exceeded 6%; this demonstrates that the RSM model accurately predicts the Qe values for the four studied HMs using the modified LDO adsorbent.

### 3.2. Characterization of the Modified LDO and LDH Sorbents

#### 3.2.1. Influence of pH_pzc_

**Figure 6:**
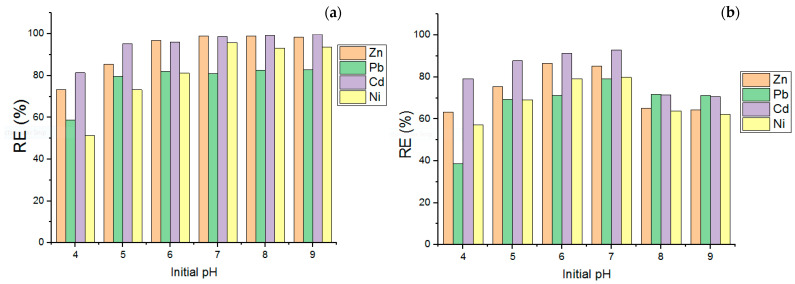
Impact of initial solution pH on the RE% of Zn, Pb, Cd, and Ni by modified LDO (**a**) and LDH sorbent (**b**).

**Figure 7:**
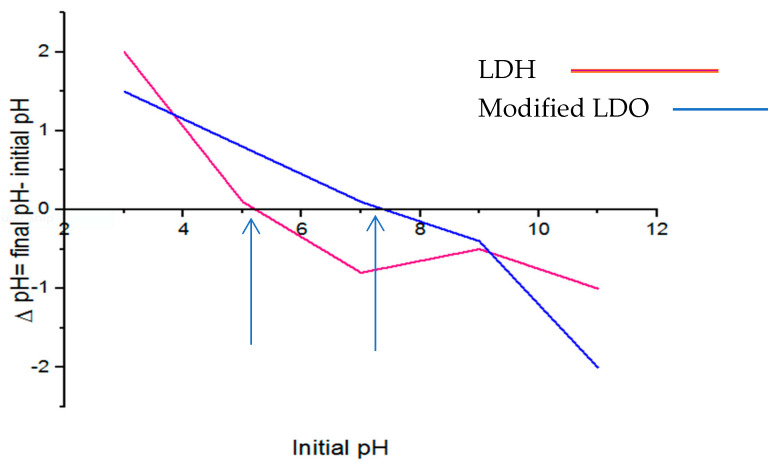
pH_pzc_ of the modified LDO and LDH sorbents.

One of those most critical factors is pH, which is powerful in HMs sorption. It is closely related to metal and hydrogen ions for competition. The influence of the solution’s initial pH on the RE% of Zn, Pb, Cd, and Ni was evaluated at various ranges of pH values ~(4.0 to 9.0) onto the modified LDO and LDH sorbents, as shown in Figure 6. In modified LDO, the maximum RE % of Zn and Pb was obtained at pH 8, while Cd and Ni exhibited the maximum RE% at pH 7. However, the highest value of RE% for four studied HMs by LDH adsorbent was about 7. The RE% of HM by modified LDO adsorbent increased with increasing pH from 4 to 7 and, after that, did not change and reach equilibrium. Also, the value of RE% by LDH adsorbent after pH 7 decreased with increased pH. At pH 4.0 to 7.0, weak sorption of HMs ions related to competition with H+ ions for active sites of the modified LDO and LDH sorbents [26]. At a range of 7 to 8, the electrostatic repulsions between the surface of modified LDO and the positively charged Zn, Pb, Cd, and Pb are depressed, which increases the RE% of HMs. The decrease in RE (%) of HMs at pH values (7.0–9.0) could be related to the repulsion between the negative charge of the anionic species in solution and the negative surface charge of the undesired sorbent [29]. It is easy to understand that the neutralization condition is favorable for HMs sorption. Meanwhile, this result indicates that modified LDO adsorbent maintains a stable adsorption performance under neutralization and basic environment. As a result, the subsequent experiments were carried out at about 7. Based on Figure 7, the measured value pH_PZC_ for the modified LDO and LDH sorbents was ~7 and 5. This result indicated that at pH below pHpzc, modified LDO becomes positively charged, and at pH bigger than pHpzc, it becomes negatively charged. Some researchers have reported that at pH values below pHpzc, the removal efficiency of HMs increases slightly. This phenomenon is related to the saturation of the sorbent surface by H+ ions or the presence of electrostatic repulsion between positively charged HMs. If the pH value is greater than pH_pzc_, the surface of the sorbent might become negatively charged, leading to a higher force of attraction for cationic species and resulting in increased sorption [30]. It follows that it can be justified that this parameter is one of the most crucial in improving the RE value of HMs.

#### 3.2.2. FTIR

**Figure 8:**
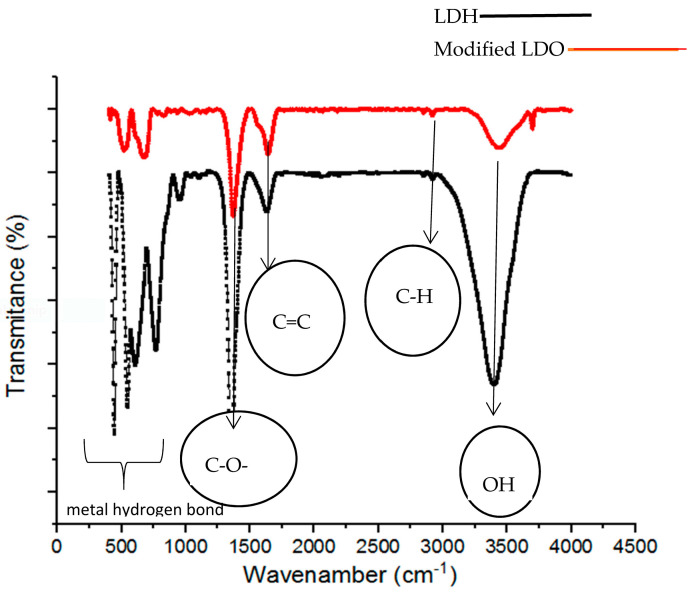
IR Spectra presented between 4000 and 400 wavenumbers for analysis of the modified LDO and LDH sorbents.

The functional groups of the modified LDO and LDH sorbents were assessed using FTIR analysis (Figure 8). According to the results, the modified LDO adsorbent exhibited a greater number of functional groups compared to the LDH sorbent. Despite some similarities between the two adsorbents, their distinctions were linked to the presence of pectin and their calcination temperatures. The peaks at 3500, 3000, 1600, and 1350 cm^−1^ were indicative of –OH, C-H, C=C, and C-O-C functionalities, respectively [22,27]. The band between 400 to 800 cm^−1^ was appeared in the modified LDO and LDH sorbents was attributed to the lattice vibrations for Mg-O and Al-O [31]. The range of 3500–3700 cm^−1^ was associated with residual water molecules and hydroxyl groups. In the modified LDO, two peaks were observed within this range, while only one peak existed in the LDH adsorbent. A reduction in the intensity of the absorption peak at 1358 cm^−1^ (specific O-C-O stretching vibration) was observed in the modified LDO adsorbent compared to the LDH sorbent. This decrease indicated that during the temperature-based process (calcination process), the interlayer carbonate anion groups underwent disintegration [27,31].

#### 3.2.3. Reusability of the Modified LDO and LDH Sorbents for HMs Adsorption

**Figure 9:**
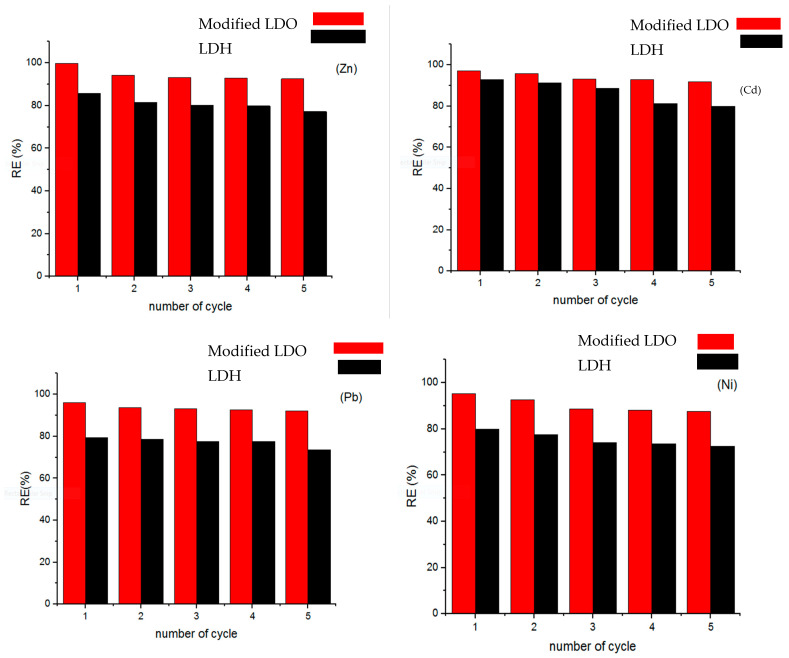
Removal efficiency (%) of HMs by the modified LDO and LDH sorbents up to five consecutive cycles.

A crucial aspect of any sorbent is its reusability, which contributes to the economic viability of the sorption process [32]. In this study, the reusability of the modified LDO and LDH sorbents was investigated across five consecutive cycles (Figure 9). The removal efficiency (RE) (%) of HMs using both the modified LDO and LDH sorbents was evaluated at initial Zn, Pb, Cd, and Ni concentrations of 100 mg L^−1^, an adsorbent concentration of 1.0 g L^−1^, and a reaction time of 180 min. For instance, the results demonstrated that the RE (%) of Cd using the modified LDO adsorbent decreased from 97% to 91%, while for the LDH adsorbent, it decreased from 92% to 79%; this highlights the successful and sustained sportive application of the modified LDO sorbent over five consecutive cycles, underscoring that modified LDO (with a molar ratio of 3:1, 600 °C, and 7% pectin) possesses high durability and robust mechanical strength. Conversely, the LDH adsorbent (with a molar ratio 2:1) exhibited instability and reduced efficiency during testing. Therefore, while the LDH adsorbent (simple LDH) is not a suitable candidate for purifying aqueous solutions, the modified LDO adsorbent proves to be an excellent choice for removing HMs.

#### 3.2.4. Morphology

**Figure 10:**
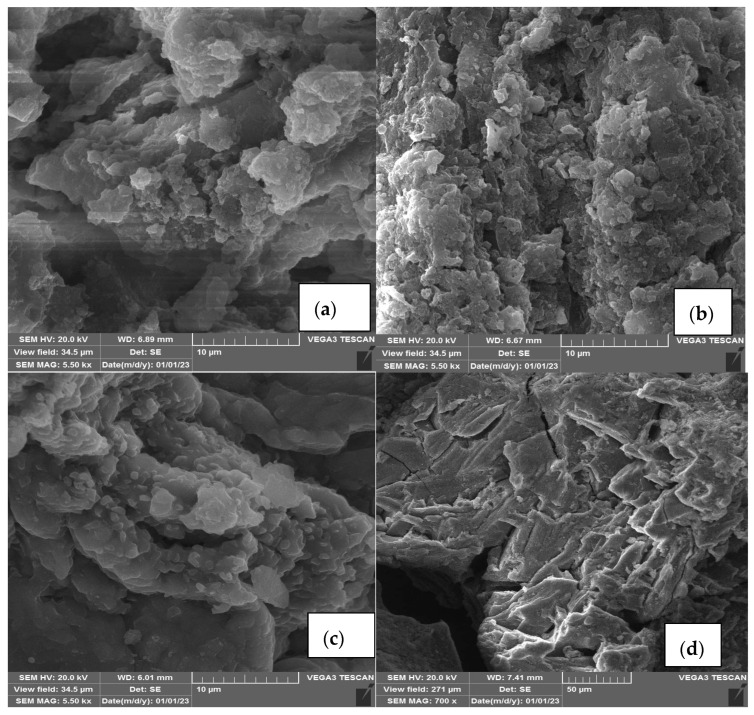
SEM image of modified LDO adsorbent before (**a**) and after adsorption (**b**), LDH adsorbent before (**c**) and after adsorption process (**d**).

The surface morphology of both the modified LDO and LDH sorbents was examined through SEM–EDX analysis (Figure 10, Table 8). Figure 10 showed that the modified LDO adsorbent displayed more porous structures and an irregular, rough shape, whereas the LDH adsorbent exhibited a smooth surface. The modified LDO sorbent featured rough surfaces, a porous structure, and an irregular shape, which facilitated the creation of active sites for the sorption of HMs onto the adsorbent [33,34]. Following the adsorption experiment, both adsorbents underwent deformation, manifesting as the formation of a white layer, a reduction in cavities, and the appearance of cracks, indicative of the successful sorption of HMs onto the sorbent [35]. The EDX analysis outcomes for the modified LDO and LDH sorbents revealed the presence of fundamental elements such as C, O, Al, and Mg. The modified LDO adsorbent contained higher levels of C, Al, and Mg, attributed to the elevated cation ratio (Mg:Al 3:1) and the ratio of pectin to LDO when compared to the LDH adsorbent (Mg:Al 2:1). Post adsorption experiment, the quantities of Zn, Pb, Cd, and Ni increased in both the modified LDO and LDH sorbents adsorbents, although the increase was more pronounced in the modified LDO adsorbent. The sequence of HMs amounts in the modified LDO adsorbent was Zn > Pb > Ni > Cd (refer to Table 5). Consequently, based on the SEM–EDX findings, it is likely that the modified LDO sorbent possessed a higher Qe than the LDH sorbent. The results of BET analysis showed that LDH sorbent had a low surface area (7.06 m^2^ g^−1^) and a small pore size (5.22 nm). While modified LDO sorbent had a high surface area (116 m^2^ g^−1^) and pore size (16 nm).

### 3.3. Adsorption Kinetics and Isotherms of Modified LDO Sorbent

**Figure 11:**
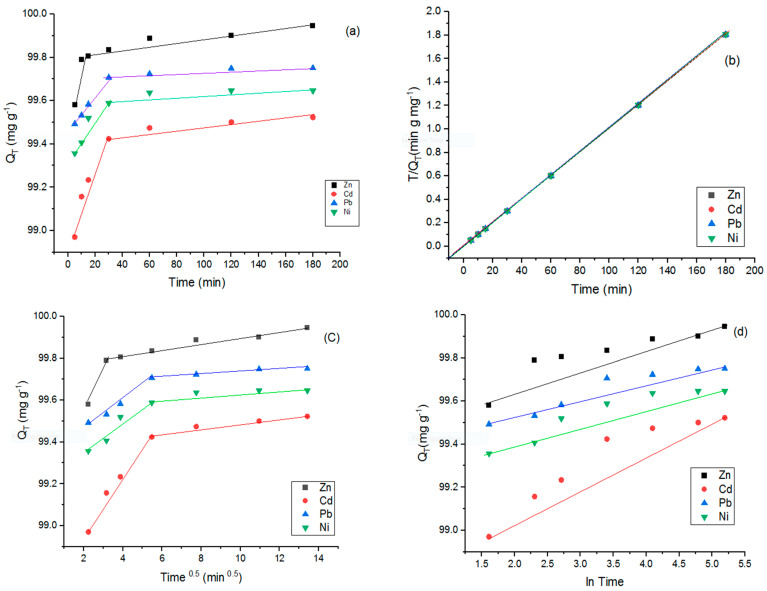
Adsorption of HMs by modified LDO Adsorbent at different contact times (5–180 min) (**a**), adsorption kinetics of HMs onto modified LDO adsorbent: pseudo-second-order (**b**), intraparticle diffusion (**c**), and Elovich (**d**). Initial HMs concentration (C0) = 180 mg L^−1^, temperature (T) = 25 °C, adsorbent dose = 1 g L^−1^, and contact time = 5–180 min.

To evaluate the rate-controlling step of HMs by the sorbent, adsorption kinetics serves as a valuable tool [36,37]. In order to determine the kinetic mechanism of Zn, Cd, Pb, and Ni adsorption onto the modified LDO (with a molar ratio of cation: 3:1, ratio of pectin to LDO: 7% *w*/*w*, and temperature: 577 °C), various kinetic models under Initial HMs concentration (C_0_) = 180 mg L^−1^, temperature (T) = 25 °C, adsorbent dose = 1 g L^−1^, Contact Time = 180 min condition were employed. The determination of kinetic model parameters was conducted using linear regression analysis, considering high R^2^ values and low SE values (Table 9, Figure 11).

As evident from Figure 11, the Qe values for Zn, Cd, Pb, and Ni increase with an extended reaction time (5–180 min). This trend can also be explained by the notion that a high concentration of HMs creates a robust driving force, facilitating ion diffusion from the solution phase to the modified LDO sorbent. Additionally, it enhances the frequency of collisions between the sorbates and the active sites of the sorbent [38].

The Qe values increased up to 60 min (except for Zn), after which they reached equilibrium and showed no further change. The specific timing of these two phases and when they occurred was indicative of the type of HMs present [19]. The initial phase, characterized by rapid sorption rates, lasted approximately 60 min for Ni, Cd, and Pb. In contrast, this initial phase was shorter for Zn, lasting around 30 min.

**Figure 12:**
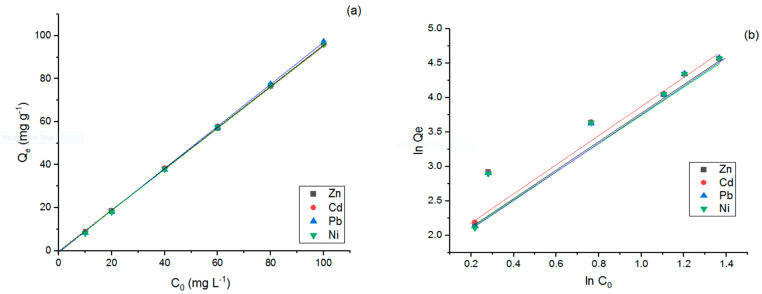
Effect of initial concentration on adsorption capacity (**a**) and Freundlich plot for HMs adsorption onto modified LDO adsorbent (**b**). Initial HMs concentration (C_0_) = 10–100 mg L^−1^, temperature (T) = 25 °C, adsorbent dose = 1 g L^−1^, and total contact time = 180 min.

The initial rapid sorption stage was associated with the sorbent’s external surface [35], wherein an abundance of adsorption sites existed, and no inner diffusion limitations were present [39]. The swift achievement of equilibrium in HM sorption indicated the rapid removal of HMs from the aqueous solution [40]. The quick phase at the outset was succeeded by equilibrium due to a reduction in functional group sites [41]. This fast sorption phase denotes strong electrostatic attractions between HMs and the sorbent, while the subsequent slow/equilibrium phase signifies that the active sites on the sorbent’s surface are filled or saturated by HMs [14]. Based on the observations in Figure 10, the order of Qe values for the modified LDO adsorbent in various aqueous solutions containing different HMs generally followed the sequence Zn > Pb > Ni > Cd. This outcome confirmed the EDX results. The Qe value of the modified LDO adsorbent in this study was greater than that of other sorbents used for removing various pollutants, such as Zn-Fe-LDH (74.50 mg g^−1^) [3], Mn-Al-LDO/biochar (66 mg g^−1^) [42], and Chitosan-PC (32.58 mg g^−1^) [43]. According to Table 9 and Figure 12, the laboratory data were best fitted by the pseudo-second-order model (with R^2^ of 1 and SE of 0.01 for Zn and R^2^ of 1 and SE of 0.007 for Pb) and the Elovich model (with R^2^ of 0.95 and SE of 0.44 for Cd, and R^2^ of 0.93 and SE of 0.49 for Ni). These models exhibited better fitting than other models.

Also, Qe exp was closer to Qe cal, demonstrating that the best model was a pseudo-second-order equation [44]. Due to this result, the sorption of Zn and Pb is mostly controlled by the chemisorption phenomenon via a chemical reaction between the sorbents and the sorbates [45]. The value of k_2_ was 0.0007, 0.0005, and 0.001 (g mg^−1^ min^−1^) for Zn, Ni, Pb, and Cd, respectively. These outcomes show that the sorption rate of HMs was in the order of Cd > Zn > Ni = Pb.

Moreover, the Elovich model showed that the sorption process of Cd and Ni onto the modified LDO was heterogeneous [46].

Figure 12 shows that the Zn Cd, Pb, and Ni plots were divided into two steps. In the initial phase, four studied HMs were absorbed onto the outer surfaces of the sorbent. Then, in the second phase, the HMs diffused into pores and inner surfaces of sorbents (This phenomenon is called intraparticle diffusion) [47]. The final phase of the plot shows the sorption of HMs at equilibrium [48]. In general, the rate of sorption of HMs onto sorbent was controlled by two mechanisms: internal and external diffusion processes.

The influence of initial HMs concentration on the Qe (mg g^−1^) and RE (%) of the modified LDO sorbent was investigated (see Figure 11a, Table 9). The Qe values for Zn, Pb, Cd, and Ni increased from 8.75 to 96.07, 8.45 to 97.26, 8.91 to 96.07, and 8.22 to 96.02 mg g^−1^, respectively, as the concentration of HMs rose from 10 to 100 mg L^−1^; this indicates that the modified LDO proves to be an efficient sorbent for the sorption of the four studied HMs in contaminated solutions, even at concentrations of up to 100 mg L^−1^. Additionally, the enhancement in Qe for the modified LDO sorbent can be attributed to the simplified contact between the HMs and the active sites of the sorbent. The highest mean RE (%) was achieved for Cd. This outcome underscores that the adsorbent’s capability for HMs removal is not only dependent on the factors affecting the produced adsorbent but also strongly influenced by the type of metal.

According to reports from some researchers, the reduction in the value of RE (%) as the concentration of HMs increases is often attributed to the competition between HMs and binding sites on the active sites of the sorbent [49]. However, contrary to certain studies, the present research observed an increase in the RE (%) process. This contrasting result can be attributed to the abundance of active sites and their unsaturation [50].

The adsorption isotherm process provides crucial data that can unveil the mechanism of the adsorption process and shed light on the interactions between sorbate molecules and the surface of the sorbent [51].

Freundlich model was chosen as the excellent equation to explain the behavior of HMs sorption because of its comparatively high R^2^ and low SE values (Table 10). The Freundlich adsorption isotherm had a linear shape, as shown in Figure 12; it is possible to calculate the k_f_ and 1/n in, which both provide useful information. When the parameter n is between 1 and 10 (1/n less than 1), the sorbent’s surface is heterogeneous, and Zn, Cd, Pb, and Ni are well-suited to the sorption process [52].

### 3.4. Comparison Adsorption Capacity of Modified LDO toward Other Agricultural Sorbents for HMs Removal

Since the adsorption was taken place under different operation conditions, the Qe is not true. However, modified LDO sorbent shows greater Qe in comparison to most agricultural sorbents (Table 11).

## 4. Conclusions

This study focused on evaluating the efficiency of BBD–RSM and Taguchi approaches in optimizing the synthesis conditions of LDH/LDO as a new sorbent for removing Zn, Cd, Pb, and Ni from aqueous solutions. The three parameters considered were the molar ratio of cation, the ratio of LDO/LDH to pectin, and the temperature. The results demonstrated that the modified LDO (LDO-PC composites) prepared under optimal conditions exhibited high efficiency, active sites, irregular shape stability, abundant functional groups, and more cavities compared to other preparation conditions. As reported in previous studies, the Qe of the modified LDO exceeded that of the LDO synthesized using traditional methods. The most influential factors affecting the adsorption capacity were ranked as C > B > A for Zn, B > A for Cd, and B > A > C for Pb and Ni. These findings suggest specific LDO preparation conditions tailored to each HM’s removal might be necessary. The type of metal and the sorbent’s synthesis conditions emerged as the two most critical parameters influencing the RE% of HMs. The experimental data aligned well with the mathematical model, indicating the suitability of RSM. The pseudo-second-order (Initial HMs concentration (C_0_) = 180 mg L^−1^, temperature (T) = 25 °C, adsorbent dose = 1 g L^−1^, contact Time = 5–180 min) and Freundlich equations (Initial HMs concentration (C_0_) 10–100 mg L^−1^, T = 25 °C, adsorbent dose = 1 g L^−1^, contact time = 180 min) provided the best descriptions for the sorption kinetics and isotherms, respectively. Even after undergoing five cycles of recycling, the RE% of HMs remained above 90%. The main mechanisms behind the adsorption of HMs onto the modified LDO sorbent were electrostatic attraction and chemisorption. In conclusion, the modified LDO represents a promising, eco-friendly, cost-effective, and reusable adsorbent for removing HMs from wastewater. The research outcomes underscored the high predictive potential of both Taguchi and RSM approaches for the adsorption capacity of metals. When comparing these two statistical methods, it becomes evident that RSM offers greater accuracy and considers more interactions, while Taguchi optimizes the number of tests and proves more cost-effective than RSM.

## Figures and Tables

**Table 1:** Summary of the experimental variables.

Variable	Unit		Range and Level	
		−1	0	1
A: molar ratio (Mg:Al)	-	2	3	4
B: ratio of pectin to LDO	*w*/*w*%	0	5	10
C*:* temperature	RT	0	300	600

**Table 2:** BBD design matrix for the independent variables.

Run	Factor A	Factor B	Factor C
1	2	0	300
2	4	0	300
3	2	10	300
4	4	10	300
5	2	5	0
6	4	5	0
7	2	5	600
8	4	5	600
9	3	0	0
10	3	10	0
11	3	0	600
12	3	10	600
13	3	5	300
14	3	5	300
15	3	5	300

**Table 3:** Taguchi design matrix for the independent variables.

	Factor 1	Factor 2	Factor 3
Run	A: Molar Ratio	B: Pectin/LDO	C: Temperature
1	Level 2 of A	Level 1 of B	Level 1 of C
2	Level 2 of A	Level 2 of B	Level 2 of C
3	Level 2 of A	Level 3 of B	Level 3 of C
4	Level 1 of A	Level 1 of B	Level 2 of C
5	Level 1 of A	Level 2 of B	Level 3 of C
6	Level 2 of A	Level 3 of B	Level 1 of C
7	Level 3 of A	Level 2 of B	Level 3 of C
8	Level 3 of A	Level 2 of B	Level 1 of C
9	Level 3 of A	Level 3 of B	Level 2 of C

**Table 4:** BBD design matrix and actual responses for Qe (mg g^−1^) and RE (%) values.

	Qe (mg g^−1^)				RE (%)			
Run	Zn	Cd	Pb	Ni	Zn	Cd	Pb	Ni
1	90.975	83.304	90.648	89.216	90.975	83.304	90.648	89.216
2	92.944	99.865	91.516	92.674	92.944	99.865	91.516	92.674
3	99.207	96.18	95.74	96.238	99.207	96.18	95.74	96.238
4	99.817	95.76	96.845	98.111	99.817	95.76	96.845	98.111
5	90.663	95.31	93.665	94.81	90.663	95.31	93.665	94.81
6	91.865	96.436	94.85	96.657	91.865	96.436	94.85	96.657
7	98.867	98.839	99.839	95.918	98.867	98.839	99.839	95.918
8	99.986	99.892	99.79	99.82	99.986	99.892	99.79	99.82
9	84.208	91.179	88.629	88.187	84.208	91.179	88.629	88.187
10	97.942	97.412	99.607	91.83	97.942	97.412	99.607	91.83
11	98.225	93.59	98.849	91.374	98.225	93.59	98.849	91.374
12	99.996	99.877	99.747	99.884	99.996	99.877	99.747	99.884
13	99.873	99.196	99.486	97.792	99.873	99.196	99.486	97.792
14	99.969	97.904	99.659	97.898	99.969	97.904	99.659	97.898
15	99.965	98.969	99.623	98.911	99.965	98.969	99.623	98.911

**Table 5:** Taguchi design matrix for the three independent variables with the actual responses for Qe (mg g^−1^).

Run	Qe of Zn	Qe of Cd	Qe of pb	Qe of Ni
1	84.2	91.179	88.629	88.187
2	90.97	83.304	90.648	89.216
3	97.94	97.412	99.607	91.83
4	98.86	98.839	99.839	95.918
5	91.86	96.436	94.85	96.657
6	99.87	99.196	99.486	97.792
7	99.81	95.76	96.845	98.111
8	99.98	99.892	99.79	99.82
9	99.99	99.877	99.747	99.884

**Table 6:** The rank of variables based on RSM and Taguchi.

Zn	A	B	C
Delta	6.68	7.74	1.92
Rank-Taguchi	2	1	3
Rank-RSM	3	1	2
Cd			
Delta	5.74	4.98	2.75
Rank-Taguchi	1	2	3
Rank-RSM	1	2	3
Pb			
Delta	4.20	5.38	1.13
Rank-Taguchi	2	1	3
Rank-RSM	3	1	2
Ni			
Delta	7.52	4.45	0.53
Rank-Taguchi	1	2	3
Rank-RSM	2	1	3

**Table 7:** Verification of adsorption model.

Type of Adsorbent	Molar Ratio	The Ratio of Pectin to LDO (*w*/*w*%)	Temperature (RT)	Qe of Zn (mg g^−1^)	Qe of Cd (mg g^−1^)	Qe of Pb (mg g^−1^)	Qe of Ni (mg g^−1^)
Modified LDO (RSM)	3.012	6.986	577.355	101.518	100.861	101.962	99.913
LDH (RSM)	2.016	0.000	0.007	81.357	83.575	85.390	87.696
Modified LDO (Taguchi)	3	6	600	99.990	95.766	96.605	95.268
LDH (Taguchi)	3	0	0	84.200	95.766	96.605	95.268

**Table 8:** The EDX analysis of modified LDO and LDH sorbents adsorbent before and after the adsorption process.

	W% of Element before Adsorption		W% of Element after Adsorption	
Element	LDH Adsorbent	Modified LDO Adsorbent	LDH Adsorbent	Modified LDO Adsorbent
C	20.53	21.04	23.81	17.83
O	52.25	46.15	54.26	48.82
Mg	17.11	23.38	10.18	13.7
Al	8.25	8.37	3.1	2.09
Ni	0.69	0.24	2.91	3.61
Zn	0.16	0.28	2.1	5.85
Cd	0.48	0.26	1.16	1.95
Pb	0.53	0.28	2.48	6.15

**Table 9:** Adsorption kinetic constants of HMs adsorption.

		Pseudo-Second Order					Intraparticle Diffusion			Elovich			
Metal Type	Qe _(exp)_	K_2_	Qe _(cal)_	R^2^	SE	k_3_	c	R^2^	SE	α	β	R^2^	SE
Zn	99.94	0.0003	100	1	0.017	0.023	99.66	0.81	0.07	99.54	0.081	0.9	0.02
Pb	99.75	0.0002	100	1	0.005	0.022	99.47	0.87	2.2	99.37	0.078	0.95	0.43
Cd	99.52	0.0004	100	0.75	0.15	0.04	99.03	0.85	2.36	98.80	0.151	0.95	0.44
Ni	99.64	0.0002	100	0.74	0.48	0.024	99.38	0.84	2.48	99.25	0.084	0.93	0.49

**Table 10:** Parameters of Freundlich isotherm model for HMs adsorption onto modified LDO adsorbent.

Metal	$\frac{1}{n}$	$k_{f}$	R^2^	SE
Zn	0.54	8.24	0.97	0.12
Pb	0.271	2.05	0.97	0.06
Cd	0.566	11.13	0.94	0.18
Ni	0.381	3.380	0.88	0.16

HMs C_0_ = 10–100 mg L^−1^, T = 25 °C, adsorbent dose = 1 g L^−1^, contact time = 180 min.

**Table 11:** Comparison adsorption capacity (mg g^−1^) of other sorbents.

Sorbent	Qe (mg g^−1^)	Concentration (mg L^−1^)	Metal	Contact Time (min)	Adsorbent Dose (g L^−1^)	References
Chitosan–Pectin	97	100	Pb	60	0.83	[53]
LDH/biochar	24	50	Cd	300	4	[54]
MgAl-LDO	19.02	100	Ni	1220	1	[55]
manure biochar	19.8	10	Cu	1440	0.5	[56]
MgAl-LDO-PC	99.94	100	Zn	180	1	Present research
MgAl-LDO-PC	99.75	100	Pb	180	1	Present research
MgAl-LDO-PC	99.52	100	Cd	180	1	Present research
MgAl-LDO-PC	99.64	100	Ni	180	1	Present research

## Data Availability

The dataset analyzed is available from available from the corresponding author on reasonable request.

## Supplementary Materials

The following supporting information can be downloaded at: https://www.mdpi.com/article/10.3390/polym15183778/s1, Table S1. ANOVA results of adsorption capacity for RSM Model; Table S2. ANOVA results of adsorption capacity for Taguachi Model.
