# Supplementary material for: Optimization of LDO-Pectin Synthesis Conditions for the Removal of Metals from Wastewater: A Comparison of Response Surface Methods and Taguchi Approaches
Source: Polymers (Basel). 2023 Sep 15;15(18):3778. doi: 10.3390/polym15183778 (PMC10537719; doi:10.3390/polym15183778)
Supplement: Supplementary file 1 [file polymers-15-03778-s001.zip › polymers-2588448-supplementary.pdf]

Table S1. ANOVA results of adsorption capacity for RSM Model

	Adsorption capacity											
Source	Sum of Squares	df	Mean Square	F Value	p-value Prob > F		Sum of Squares	df	Mean Square	F Value	p-value Prob > F	
Model $_{Cd}$	240.27	9	26.70	4.83	0.0488	Model $_{Pb}$	208.33	9	23.15	263.08	<0.0001	significant
Model $_{Zn}$	337.96	9	37.55	4440.33	< 0.0001	Model $_{Ni}$	193.78	9	21.53	15.67	0.0037	significant
A $_{Cd}$	41.95	1	41.95	7.59	0.0401	A $_{Pb}$	1.21	1	1.21	13.73	0.0139	
A $_{Zn}$	3.00	1	3.00	354.89	< 0.0001	A$_{Ni}$	15.35	1	15.35	11.17	0.0205	
B $_{Cd}$	56.66	1	56.66	10.25	0.0239	B $_{Pb}$	62.14	1	62.14	706.30	<0.0001	
B $_{Zn}$	117.12	1	117.12	13849.50	< 0.0001	B$_{Ni}$	75.72	1	75.72	55.11	0.0007	
C $_{Cd}$	17.59	1	17.59	3.18	0.1345	C $_{Pb}$	57.64	1	57.64	655.12	<0.0001	
C $_{Zn}$	131.19	1	131.19	15512.80	< 0.0001	C$_{Ni}$	30.08	1	30.08	21.89	0.0054	
AB $_{Cd}$	72.09	1	72.09	13.04	0.0154	AB $_{Pb}$	0.014	1	0.014	0.16	0.7060	
AB $_{Zn}$	0.46	1	0.46	54.60	0.0007	AB$_{Ni}$	0.63	1	0.63	0.46	0.5290	
AC $_{Cd}$	1.332E-003	1	1.332E-003	2.411E-004	0.9882	AC $_{Pb}$	0.38	1	0.38	4.33	0.0921	
AC $_{Zn}$	1.722E-003	1	1.722E-003	0.20	0.6707	AC$_{Ni}$	5.92	1	5.92	0.77	0.4208	
BC $_{Cd}$	7.290E-004	1	7.290E-004	1.319E-004	0.9913	BC $_{Pb}$	25.40	1	25.40	288.70	<0.0001	
BC $_{Zn}$	35.78	1	35.78	4230.75	< 0.0001	BC$_{Ni}$	5.92	1	5.92	4.31	0.0925	
A^2 $_{Cd}$	7.28	1	7.28	1.32	0.3031	A^2 $_{Pb}$	28.68	1	28.68	325.97	<0.0001	
A^2 $_{Zn}$	14.38	1	14.38	1700.83	< 0.0001	A^2_{Ni}	0.023	1	0.023	0.017	0.9020	
B^2 $_{Cd}$	45.45	1	45.45	8.22	0.0351	B^2 $_{Pb}$	35.83	1	35.83	407.21	<0.0001	
B^2 $_{Zn}$	18.30	1	18.30	2163.85	< 0.0001	B^2_{Ni}	60.91	1	60.91	44.33	0.0012	
C^2 $_{Cd}$	0.41	1	0.41	0.074	0.7961	C^2 $_{Pb}$	0.20	1	0.20	2.29	0.1905	
C^2 $_{Zn}$	25.28	1	25.28	2989.55	< 0.0001	C^2_{Ni}	6.43	1	6.43	4.68	0.0828	
Lack of Fit $_{Cd}$	26.68	3	8.89	18.69	0.0512	Lack of Fit $_{Pb}$	0.42	3	0.14	16.93	0.068	not significant
Lack of Fit $_{Zn}$	0.036	3	0.012	5.822E-003	3	Lack of Fit$_{Ni}$	6.11	3	2.04	5.33	0.98	not significant
Pure Error $_{Cd}$	0.95	2	0.48			Pure Error $_{Pb}$	0.017	2	8.332E-003			
Pure Error $_{Zn}$	5.899E-003	2	2.949E-003			Pure Error$_{Ni}$	0.76	2	0.38			
Cor Total $_{Cd}$	267.90	14				Cor Total $_{Pb}$	208.77	14				
Cor Total $_{Zn}$	338.00	14				Cor Total$_{Ni}$	200.65	14				
R^2 $_{Cd}$	0.89					R^2 $_{Pb}$	0.99					
R^2 $_{Zn}$	0.99					R^2_{Ni}	0.96					
Adj R^2 $_{Cd}$	0.71					Adj R^2 $_{Pb}$	0.99					
Adj R $_{Zn}$	0.99					Adj R^2_{Ni}	0.90					
Pred R^2 $_{Cd}$	0.60					Pred R^2 $_{Pb}$	0.96					
Pred R^2 $_{Zn}$	0.99					Pred R^2_{Ni}	0.50					
Adeq Precision $_{Cd}$	8.74					Adeq Precision $_{Pb}$	45.765					
Adeq Precision $_{Zn}$	209.898					Adeq Precision$_{rNi}$	12.14					

Table.S2. ANOVA results of adsorption capacity for Taguachi Model

Source	DF	Adj SS	Adj MS	F-Value	P-Value	R^2
$Regression_{Zn}$	3	209.874	69.9578	7.78	0.025	0.77
$Regression_{Cd}$	3	24.850	8.2833	0.20	0.894	0.55
$Regression_{Pb}$	3	38.830	12.9435	0.58	0.652	0.65
$Regression_{Ni}$	3	29.925	9.9751	0.39	0.765	0.31
A_{Zn}	1	0.013	0.0134	0.00	0.971	
A_{Cd}	1	0.028	0.0281	0.00	0.980	
A_{Pb}	1	0.002	0.0022	0.00	0.992	
A_{Ni}	1	8.645	8.6446	0.34	0.586	
B_{Zn}	1	85.874	85.8740	9.55	0.027	
B_{Cd}	1	20.718	20.7184	0.49	0.514	
B_{Pb}	1	30.136	30.1361	1.36	0.297	
B_{Ni}	1	8.196	8.1962	0.32	0.595	
C_{Zn}	1	60.166	60.1664	6.69	0.049	
C_{Cd}	1	1.397	1.3974	0.03	0.862	
C_{Pb}	1	0.048	0.0476	0.00	0.965	
C_{Ni}	1	0.104	0.1042	0.00	0.952	
$Error_{Zn}$	5	44.974	8.9947			
$Error_{Cd}$	5	209.783	41.9566			
$Error_{Pb}$	5	111.197	22.2394			
$Error_{Ni}$	5	127.495	25.4990			
$Total_{Zn}$	8	254.847				
$Total_{Cd}$	8	234.633				
$Total_{Pb}$	8	150.027				
$Total_{Ni}$	8	157.420				
